# CT of the medial clavicular epiphysis for forensic age estimation – raised arms position recommended

**DOI:** 10.1007/s00414-025-03521-2

**Published:** 2025-05-29

**Authors:** Saskia C Kuhnen, Martin Müller, Andreas Schmeling, Wolf-Dieter Zech, Jeremias B Klaus, Paolo Lombardo, Michael Ith, Rainer J Egli, Thomas D Ruder

**Affiliations:** 1 Institute of Diagnostic, Interventional and Pediatric Radiology InselspitaI, Bern University Hospital, University of Bern, Rosenbühlgasse 27, Bern, Bern, CH-3010 Switzerland; 2 Department of Emergency Medicine, Bern University Hospital, InselspitaI, University of Bern, Bern, Switzerland; 3 Institute of Legal Medicine, Münster, Germany; 4 Institute of Forensic Medicine, University of Bern, Bern, Switzerland; 5 Dr Kurz Roentgen Institute AG, Thun, Switzerland; 6 Radiation Protection, Image Processing Systems & Radiological Processes, Hirslanden Private Hospital Group, Zurich, Switzerland

**Keywords:** Forensic age estimation, Medial clavicular epiphysis, Computed tomography, Radiation protection, Arm positioning

## Abstract

**Objective:**

To compare the effect of arm positioning on radiation dose, scan length, and image noise in computed tomography (CT) scans of the medial clavicular epiphysis for forensic age estimation performed with the arms alongside the body (arms-down) versus elevated above the head (arms-up).

**Methods:**

Twenty consecutive CT scans were analysed, ten performed with arms-down and ten with arms-up. The scans were conducted at 120 kVp and 37 mAs reference tube current. Scan length extended from 10 mm above to 10 mm below the medial clavicular epiphysis. Dose-relevant parameters (effective CT tube current, volume CT dose index (CTDIvol), CT dose length product (DLP), and effective dose) as well as scan length and image noise were compared between arms-up and arms-down CT scans.

**Results:**

Population characteristics: 19 males, 1 female; mean weight 65.8 ± 9.2 kg; height 174.6 ± 7.8 cm; and body mass index (BMI) 21.6 ± 2.5 kg/m². Dose-relevant parameters were significantly lower with arms-up compared to arms-down (effective tube current: 80.9 ± 21.7 mAs vs. 146.0 ± 47.5 mAs, *p* = 0.001; CTDIvol: 5.5 ± 1.5 mGy vs. 9.9 ± 3.2 mGy, *p* = 0.001; DLP: 40.2 ± 13.7 mGy*cm vs. 63.8 ± 21.9 mGy*cm, *p* = 0.010; effective dose: 0.6 ± 0.2 mSv vs. 0.9 ± 0.3 mSv, *p* = 0.010). No significant differences were found in scan length, image noise, or population characteristics.

**Conclusions:**

Removing the arms from the CT beam path reduced radiation dose by 33% without affecting scan length or image noise. Given the importance of dose optimisation in non-medical examinations of potentially minor individuals, CT scans of the medial clavicular epiphysis should be performed with arms elevated above the head.

## Introduction

Over the past 20 years, the Arbeitsgemeinschaft für Forensische Altersdiagnostik (Study Group of Forensic Age Diagnostics, AGFAD) has achieved a high level in international standardisation in the process of forensic age estimation (FAE) of potentially unaccompanied minor asylum-seekers (PUMAS) [1]. This has been accomplished through the publication of procedural recommendations and organising proficiency tests among their members. Their current recommendations regarding radiologic imaging for FAE include radiographs of the left hand and the teeth to estimate bone and dental age, as well as computed tomography (CT) of the medial clavicular epiphysis (MCE) in individuals who have reached skeletal maturity of the hand and wrist [2–6].

One criticism of FAE concerns the use of ionising radiation on young individuals for non-medical purposes, particularly the use of CT, which use higher doses than conventional radiographs [7–11]. Therefore, CT dose optimisation is crucial in FAE. To address this concern, several authors recommend using a short CT scan length of 40 mm, which should achieve an effective CT dose of less than 1 millisievert (mSv), with a range from 0.2 mSv to 4.6 mSv depending on the scan protocol [4, 7,10, 12–15].

However, there is no recommendation regarding a specific arm position during CT scans of the MCE, though the default practice appears to be the arms-down position alongside the chest – likely a holdover from when age estimation was performed on radiographs of the clavicles [1, 9]. In 2021, Tozakidou et al. compared the CT parameters of MCE scans with arms-up and arms-down [9]. They observed lower image noise levels and a lower volume CT dose index in CTs performed with arms-up, but no reduction in the effective CT dose, as the scan length was longer for arms-up scans compared to those with arms-down. Nevertheless, their work revealed the potential of dose reduction by positioning the arms outside of the CT beam path.

At the Department of Diagnostic, Interventional, and Paediatric Radiology at the University Hospital in Bern (Switzerland), the scan length for MCE CTs is determined based on the scout view to avoid unnecessary exposure above and below the MCE. The CT scan starts 10 mm above and ends 10 mm below the MCE. Initially, CTs of the MCE were performed with arms-down (using a CT neck protocol). However, this practice was abandoned early on after a radiographer accidentally performed two CTs with arms-up (using a CT chest protocol), which resulted in a substantially lower radiation dose.

The primary aim of this analysis was to compare CT scan length and radiation dose parameters between CT scans of the medial clavicular epiphysis for FAE performed in two different arm positions: arms-down (alongside the body) and arms-up (elevated above the head). The secondary aim was to compare CT image noise levels between the arms-up and arms-down positions.

## Materials and methods

This analysis included dose-relevant CT data from all 20 PUMAS referred to the Department of Diagnostic, Interventional, and Paediatric Radiology at the University Hospital of Bern, Switzerland, for forensic age estimation between April and November 2019. The first 10 PUMAS were scanned with arms-down (AD), while all subsequent cases were scanned with arms-up (AU).

**Fig. 1:**
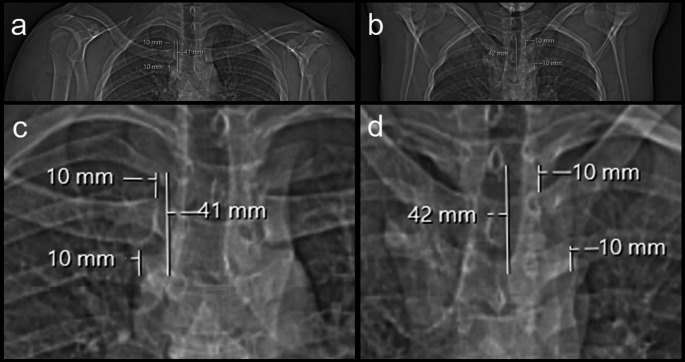
Scan length planning on scout views. Examples with arms-down (**a**) and with arms-up (**b**) as well as magnified scout views with arms-down (**c**) and arms-up (**d**) The CT scan length is planned on the magnified scout views as they allow for better delineation of cortical contour of the clavicle. The starting point of the CT scan is 10 mm above the superior contours of the medial clavicle, the endpoint is 10 mm below the inferior contour of the MCE. In these two cases, the planned scan lengths were 41 mm and 42 mm, respectively

**Fig. 2:**
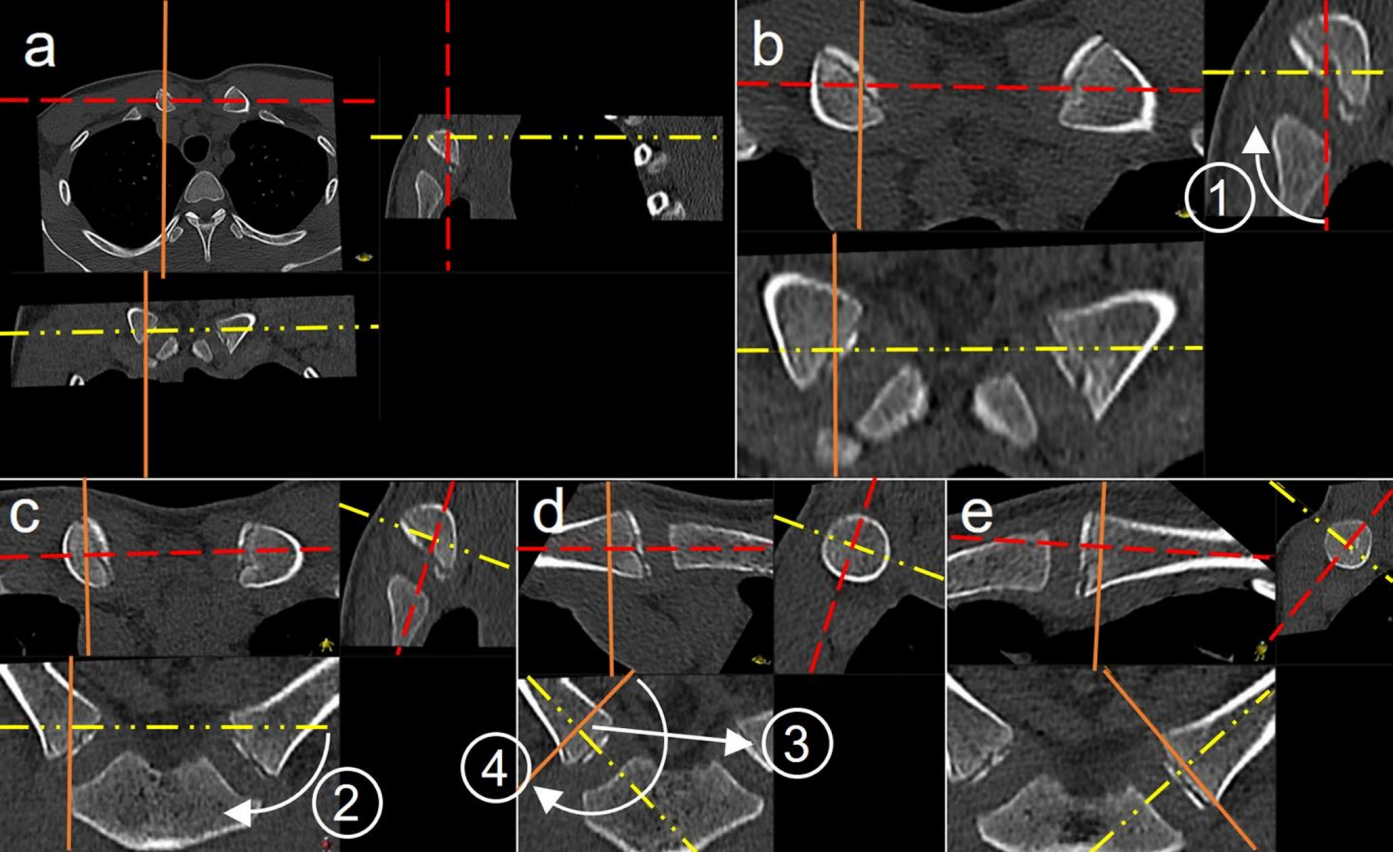
Multi-planar reconstruction of true coronal and axial views of the clavicle in MCE CT scans with arms-up. (**a**) Default position of CT images in the multi-planar reconstruction (MPR) viewer, with the crosshair centred on the right MCE. (**b**) Enlarged view of the default position. Step 1 – Adjusting to true coronal view: From the default view (**b**), rotate the red crosshair line clockwise in the sagittal view until it aligns with the clavicle centreline, as shown in (**c**). Step 2 – Generating true coronal and axial views of the right MCE: From the true coronal view in (**c**), rotate the yellow crosshair clockwise in the coronal view to align with the clavicle’s centreline, producing a true coronal and true axial view of the right MCE, as shown in (**d**). Steps 3 and 4 – Switching to true coronal and axial views of the left MCE: Move the orange and yellow crosshair lines to the left MCE. Then, rotate the orange crosshair line clockwise by 180° to achieve the orientation shown in (**e**)

Scans were performed on a 64-slice CT scanner (SOMATOM Edge, Siemens Healthineers, Erlangen, Germany) with a peak tube voltage of 120 kVp. Automatic dose modulation (CARE Dose4D, Siemens Healthineers, Erlangen, Germany) was applied to all scans, with the reference tube current (mAs ref) set to 37 mAs. CT scan length was planned based on the scout view, with the starting point 10 mm above the superior contour of the MCE and the endpoint 10 mm below the inferior contour of the MCE, as measured on the scout view by the reporting radiologist and radiographer together (Fig. 1) [9]. The field of view was coned to 200 mm for high image resolution. Scans were performed using helical image acquisition with a pitch factor of 0.6. Slice thickness was 1 mm with an increment of 0.5 mm, reconstruction kernels included a bone kernel (Br50) and a soft tissue kernel (wt40). Image analysis was performed using the hospital’s picture archiving and communication system (PACS) (IDS7, Sectra AB, Linköping, Sweden). Multiplanar reconstructions (MPR) were used to view true coronal and true axial images of each clavicle (see Fig. 2) [16].

**Fig. 3:**
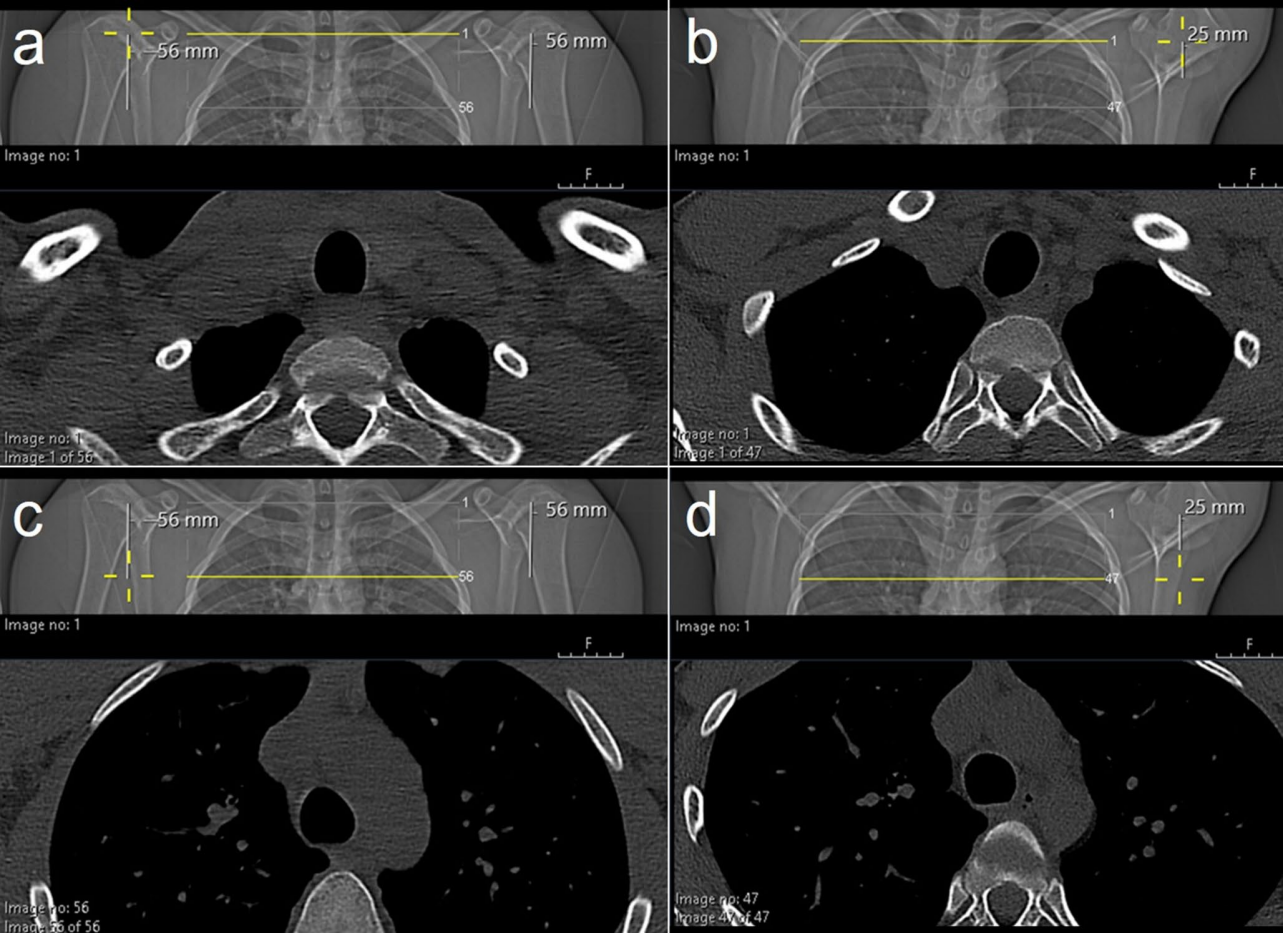
Measurement of length of humeral head/shaft within the beam path. Examples with arms-down (a)/(c) and with arms-up (b)/(d). The humeral hear/shaft length within the beam path is measured on scout views. The cross-hair tool is used to accurately determine the position on the scout view. In arms-down CTs, the measurement extends from the level of the first axial CT image (**a**) to the last axial image (**c**). In arms-up CTs, the measurement extends from the level of the first axial CT image (**b**) to the contour of the humeral head (**d**). In arms-down CTs, the length of humeral head/shaft within the beam path corresponds to the scan length. In arms-up CTs, the length of humeral head/shaft inclusion in the beam path may vary between the left and right arm. In the above presented examples, the sum humeral head/shaft included in the beam path was 112 mm for the arms-down (56 mm + 56 mm), and 25 mm for the arms-up CT (0 mm + 25 mm)

For each case, the following data were retrieved from PACS: effective CT tube current (mAs eff), volume CT dose index (CTDIvol), CT dose length product (DLP), axial CT scan length (mm), arm position during CT (i.e. arms-down or arms-up), length of humeral head/shaft within the beam path (see Fig. 3), and weight and height of the PUMAS.

The CTDIvol may be thought of as average dose per cm of a CT scan (in mGy) while the DLP represents the radiation dose for the entire scan (mGy*cm). Therefore, the CTDIvol represents a practical way of comparing radiation doses of different CT scans of the same body region irrespective of their scan length. To calculate the effective CT dose (in mSv) the DLP was multiplied by the organ-specific index for the chest (DLP [mGy*cm] × 0.0137 = effective dose [mSv]) [17, 18].

**Fig. 4:**
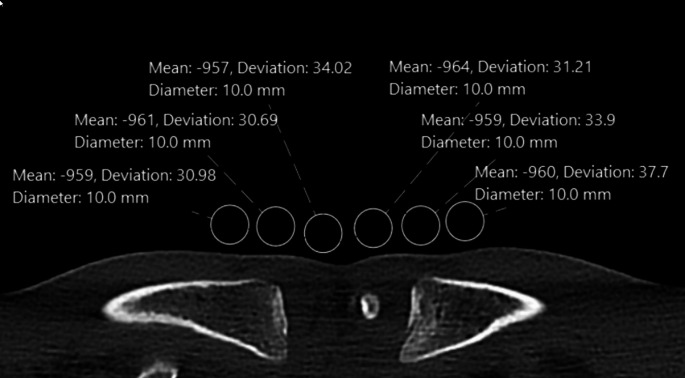
Noise measurements for air. To measure CT image noise in air, three 10 mm ROIs were placed in air, anteriorly to the right and left MCE at the midlevel of the clavicular epiphysis

**Fig. 5:**
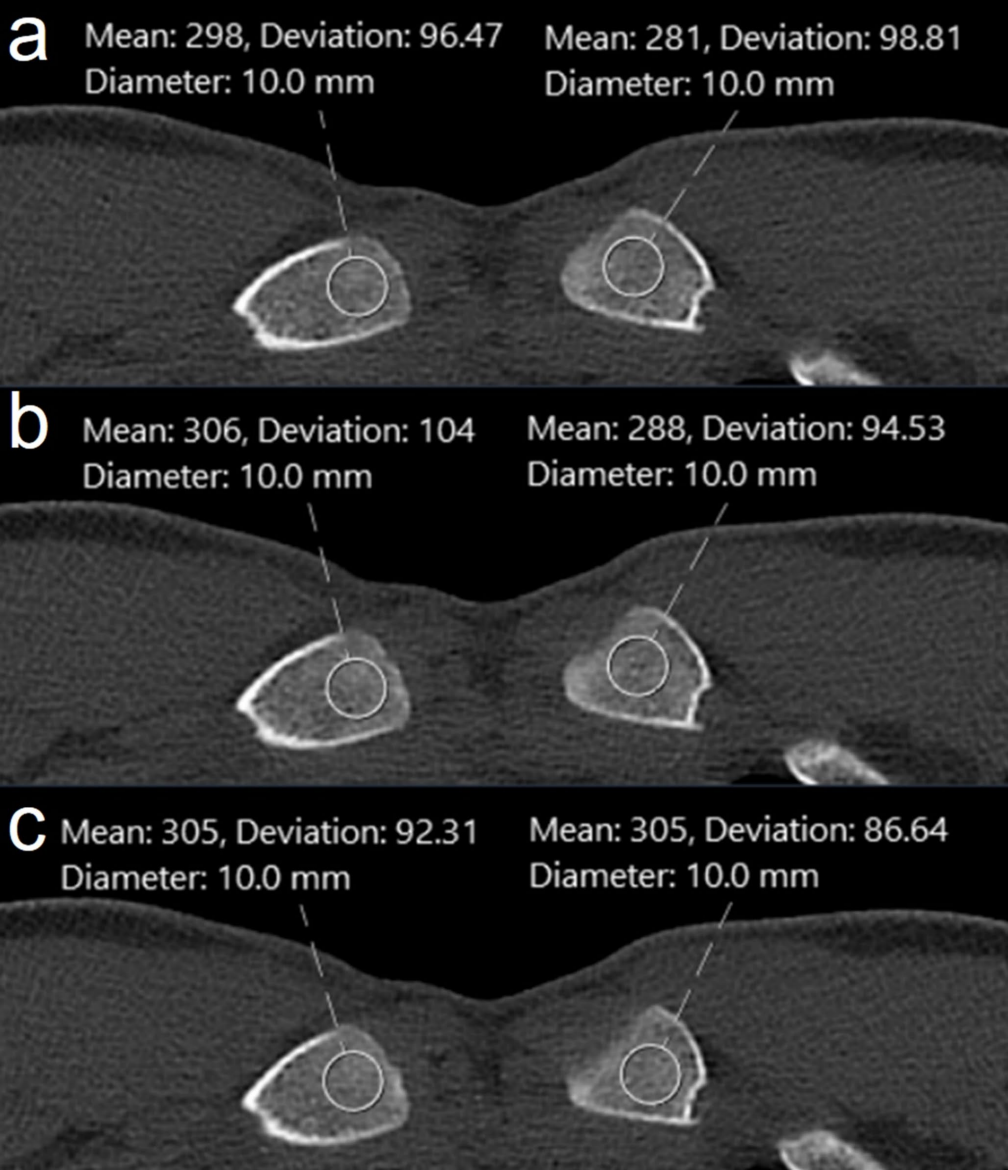
Noise measurements for bone. To measure CT image noise in bone, single 10 mm ROIs were placed within the MCE on three consecutive images of the right and left air clavicle. Great care was taken to exclude cortical bone from the ROIs

CT image noise measurements were performed by calculating the standard deviation (SD) of mean CT numbers, measured in Hounsfield units (HU), within three regions of interest (ROIs) of 10 mm on each side. For air noise measurements, the ROIs were placed anteriorly to the chest at the midlevel of the MCE [9], while for bone noise measurements, the ROIs were placed within the MCE (Figs. 4 and 5).

### Statistical analysis

The statistical analysis was performed with STATA 18.1 (StataCorp, The College Station, Texas, USA). Continuous variables were presented as mean with standard deviation (SD), with p-values calculated using an unpaired t-test. Categorical variables were reported as number (%), with p-values obtained from a Chi-squared test, respectively a Fisher’s exact test was performed if appropriate. A p-value of < 0.05 was used as the threshold for statistical significance. Cohen’s d value was calculated to quantify the effect size of the CT dose reduction between scans with arms-down and arms-up positioning. The effect size is interpreted as negligible (< 0.2), small (d = 0.2 to < 0.5), medium (d = 0.5 to < 0.8), and large (d ≥ 0.8) [19].

For graphical illustration of the comparison of the two groups, raincloud plots were created for each outcome according to the two study groups [20]. The p-value was obtained by t-tests comparing the distributions of each variable between the groups. A raincloud plot is a data visualisation tool that combines a box plot and a density plot (using STATA’s – *kdensity* – command), these two in combination are also called violin plots, as well as a strip plot.

## Results

### Population characteristics

Nineteen PUMAS were male, one was female. No significant differences were found in weight, height, and body mass index (BMI) between PUMAS scanned with arms-down and those scanned with arms-up (weight: AU 64.1 ± 7.3 kg vs. AD 67.5 ± 10.8 kg, *p* = 0.421; height: AU 173.2 ± 8.0 cm vs. AD 175.9 ± 7.7 cm, *p* = 0.461; BMI: AU 21.4 ± 2.7 kg/m2 vs. AD 21.7 ± 2.5 kg/m2, *p* = 0.786) (Table 1).

**Table 1:** Patient, scan, and outcome characteristics in the study collective and according to arm position

Variables	Total		Arm position				
	Total	(*n* = 20)	Up	(*n* = 10)	Down	(*n* = 10)	*P*-value
**POPULATION CHARACTERISTICS**							
Sex							
Female	1	[5.0]	0	[0.0]	1	[10.0]	
Male	19	[95.0]	10	[100.0]	9	[90.0]	0.305
Weight [kg]	65.8	[9.2]	64.1	[7.3]	67.5	[10.8]	0.421
Height [cm]	174.6	[7.8]	173.2	[8.0]	175.9	[7.7]	0.461
Body mass index [kg/m2]	21.6	[2.5]	21.4	[2.7]	21.7	[2.5]	0.786
**SCAN CHARACTERISTICS**							
Right humerus incl. in scan [mm]	28.4	[19.5]	12.3	[10.1]	44.5	[11.3]	< 0.001
Left humerus incl.in scan [mm]	27	[20.1]	10.2	[8.3]	43.7	[12.5]	< 0.001
Right and left humerus incl. in scan [mm]	55.3	[38.7]	22.5	[14.2]	88.2	[23.8]	< 0.001
CT tube voltage [kVp]	120	[0.0]	120	[0.0]	120	[0.0]	-
Effective CT tube current [mAs]	113.4	[49.1]	80.9	[21.7]	146	[47.5]	0.001
Reference CT tube current [mAs]	37	[0.0]	37	[0.0]	37	[0.0]	-
**RADIATION DOSES**							
CT dose index [mGy]	7.7	[3.3]	5.5	[1.5]	9.9	[3.2]	0.001
Dose length product [mGy*cm]	52	[21.5]	40.2	[13.7]	63.8	[21.9]	0.01
Effective dose [mSv]	0.7	[0.3]	0.6	[0.2]	0.9	[0.3]	0.01
CT scan length on axial MPR [mm]	50.3	[8.8]	52.1	[9.4]	48.5	[8.3]	0.377
Mean noise air [HU]	47.3	[16.5]	49.5	[19.8]	45.1	[13.2]	0.561
Mean bone air [HU]	97.6	[18.3]	99	[15.9]	96.3	[21.1]	0.749

*Note: Except for the variable sex*,* which is presented with absolute numbers followed by relative percentages in brackets*,* all other variables are described as means with standard deviations in brackets*

### CT scan characteristics

CT tube effective current was significantly lower with arms-up than with arms-down (AU 80.9 ± 21.7 mAs eff vs. AD 146.0 ± 47.5 mAs eff, *p* = 0.001) (Table 1).

**Fig. 6:**
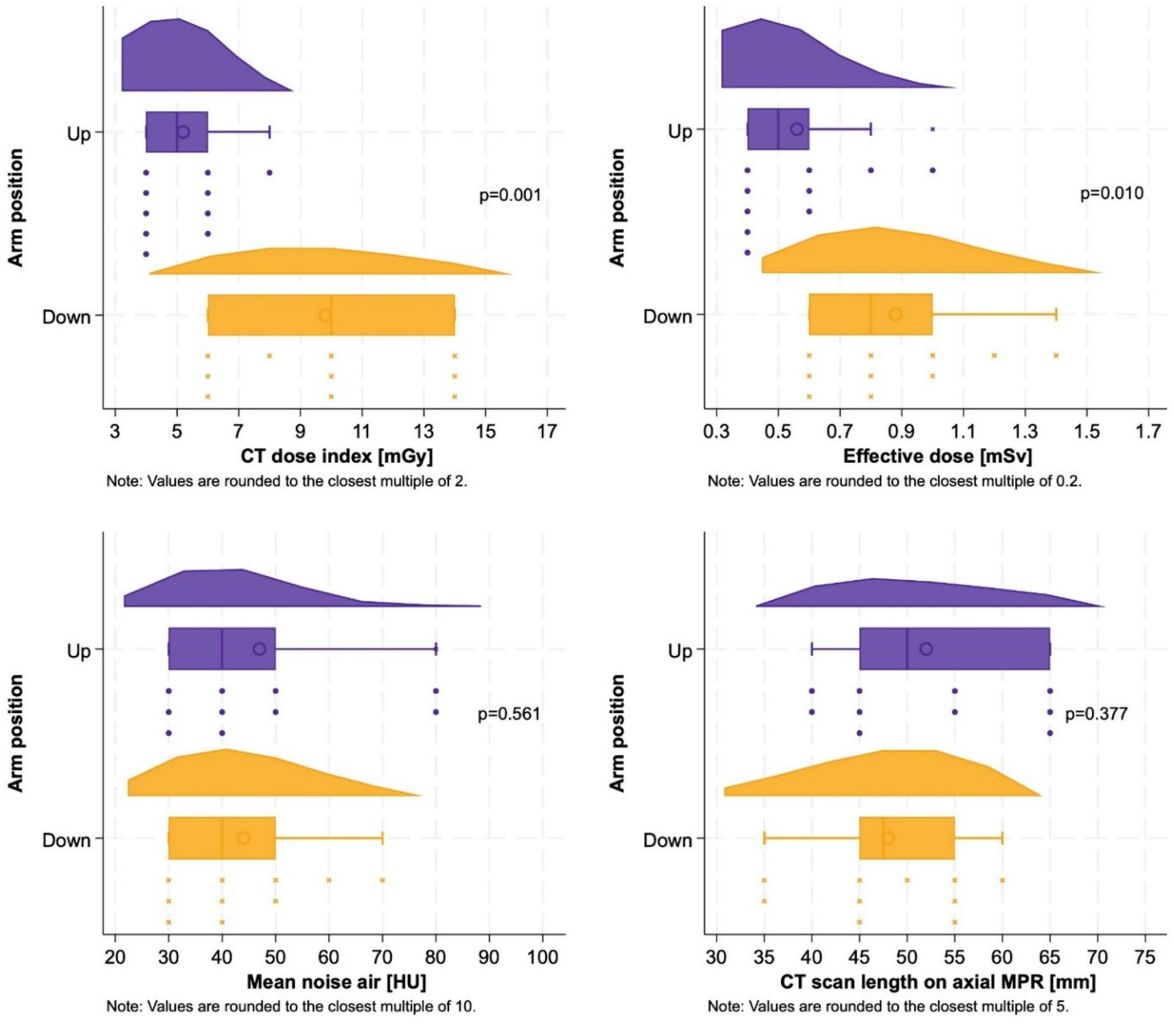
Raincloud plot (density plot, box plot, and strip plot) for the different outcomes according to the arms-up (purple) and arms-down group (yellow). Density Plot (Top): A smoothed distribution curve representing the probability density of the data. It provides insight into the overall shape of the distribution, highlighting skewness, modality (unimodal/bimodal), and spread. Box Plot (Middle): A standard horizontal box plot summarizing the central tendency and variability of the data. It displays the median (central line), interquartile range (box), and whiskers extending to the most extreme non-outlier data points. Outliers are shown as individual dots. Additionally, the mean is indicated by a hollow circle. Strip Plot (Bottom): A scatter plot of individual data points, where each filled point represents an observation. Points are stacked to reduce overplotting and improve visibility

Scan length did not significantly vary between arm positions (AU 52.1 ± 9.4 mm vs. AD 48.5 ± 8.3 mm, *p* = 0.377). The length of the humeral head and/or shaft that was included in the CT beam path was significantly shorter in scans performed with arms-up compared to arms-down (right humerus: AU 12.3 ± 10.1 mm vs. AD 44.5 ± 11.3 mm, *p* < 0.001; left humerus: AU 10.2 ± 8.3 mm vs. AD 43.7 ± 12.5 mm, *p* < 0.001; sum of right and left humerus: AU 22.5 ± 14.2 mm vs. AD 88.2 ± 23.8 mm, *p* < 0.001) (Table 1; Fig. 6).

### CT radiation dose

The volume CT dose index, CT dose length product, and effective radiation dose were significantly lower in CT scans with arms-up than with arms-down (CTDIvol: AU 5.5 ± 1.5 mGy vs. AD 9.9 ± 3.2 mGy, *p* = 0.001; DLP: AU 40.2 ± 13.7 mGy*cm vs. AD 63.8 ± 21.9 mGy*cm, *p* = 0.010; effective radiation dose: AU 0.6 ± 0.2 mSv vs. AD 0.9 ± 0.3 mSv, *p* = 0.010) (Table 1; Fig. 6). Scanning with arms-up demonstrated a large effect size (d = 1.3), indicating a substantial reduction in CT dose.

### CT image noise

There was no significant difference in image noise between CTs scanned with arms-up and with arms-down (noise air: AU 49.5 ± 19.8 HU vs. AD 45.1 ± 13.2 HU, *p* = 0.561; noise bone: AU 99.0 ± 15.9 HU vs. AD 96.3 ± 21.1 HU, *p* = 0.749) (Table 1; Fig. 6).

## Discussion

This study examined the effect of arm positioning during CT of the MCE for FAE on radiation dose, scan length, and image noise and found a 33% lower effective radiation dose in scans performed with arms-up compared to arms-down, with no significant difference in scan length or image noise.

These findings emphasise the importance of arm positioning for radiation protection, especially for age estimation, where scans are performed for non-medical reasons in young individuals. Despite the small sample size of 20 cases, the differences in dose parameters between the arms-up and arms-down groups are both statistically significant and clinically relevant. The large effect size not only supports the robustness of these results but also underscores the substantial and consistent impact of arm positioning on dose reduction. This strong effect suggests that the benefit of removing arms from the CT beam path is likely to persist in larger-scale studies.

The results also align with previous work on arm positioning for CT dose optimisation in both FAE and clinical radiology and support the practice of performing CT scans of the MCE for FAE with arms raised [9, 21–25]. The effect of arm positioning on radiation dose is particularly well-documented in trauma CT scans of the chest, abdomen, and pelvis: Brink, Karlo, and Bayer independently observed CT dose reductions of 31%, 27%, and 22%, respectively, between trauma CTs performed with arms-down and arms-up [21, 24, 25]. Positioning patients with arms-up for CT of the chest and/or abdomen has been recommended for over a decade in clinical radiology [22] More recently, Tozakidou has also demonstrated that a shoulder pull-down manoeuvre prior to CT of the c-spine results in a dose reduction of 51% [23].

Positioning the arms outside the CT beam path reduced the effective CT tube current needed for diagnostic-quality images by 44%, thus lowering the volume CT dose index from 9.9 mGy (arms-down) to 5.5 mGy (arms-up). These values align with the Swiss Federal Office of Public Health’s diagnostic dose reference values for neck CTs (12.0 mGy) and chest CTs (6.0 mGy) [17, 26]. For both of these scans, the arms are positioned outside the beam path – arms-down for neck CTs and arms-up for chest CTs. Since the sternoclavicular joints are in the chest, scanning for age estimation necessitates arms-up positioning to achieve dose levels that are within the range of the reference dose values.

The effective doses of 0.9 mSv and 0.6 mSv are equivalent to approximately 90 and 60 days of natural background radiation, or 7 and 5 standard chest radiographs, respectively [27, 28].

This study builds on Tozakidou et al.‘s work, who observed lower CTDIvol values in MCE scans with arms-up but no significant difference in radiation dose due to longer scan lengths compared to arms-down scans [9]. In this study, mean scan length was 49 mm for both CTs performed with arms-up and arms-down. This was achieved by prospectively planning the scan length on magnified scout views using the MCE’s cortical contours as landmarks as determined by the radiologist and radiographer together, ensuring consistent scan lengths across both groups (see Fig. 1).

Scanning the MCE in an arms-up position creates a steeper sternoclavicular angle, requiring separate multiplanar reconstructions (MPR) for true coronal and axial views of each clavicle [9, 29]. Current PACS systems feature MPR tools, allowing radiologists to perform these reconstructions quickly during image interpretation (see Fig. 2 for an example). While the transition from scanning with arms-down to arms-up may represent a change of habit for some practitioners, it does not alter the process of age estimation itself: The body of research on MCE ossification stages for FAE was primarily based on clinical datasets, including both CTs with arms-up and arms-down [13, 29–34].

There was no significant difference in image noise between arms-up and arms-down scans for both air and bone, despite the expectation that removing arms from the beam path would reduce noise. This finding is the result of differences in CT protocols between this study and the study of Tozakidou et al. who did observe lower noise levels with arms-up [9]. Our study used lower tube voltage and reference tube current settings (120 kVp / 37 mAs ref) than Tozakidou et al. (140 kVp / 70 mAs ref), leading to higher effective tube current through automatic modulation in our study. This increase in tube current offset the potential noise reduction from the arms-up position.

## Limitations

This study has several limitations. While the small sample size of 20 cases was sufficient to detect significant differences in radiation dose between CTs with arms-up compared to arms-down, the sample was too small for subgroup analysis to determine the influence of confounders like individual variability in body composition or partial inclusion of the humerus in arms-up scans. The authors plan to address this in a future study.

Another limitation of this single centre, single CT scanner study is that variability between CT manufacturers, scannermodels, and available CT-settings may cause other institutions to find different degrees of dose reduction when transitioning from arms-down to arms-up MCE CTs.

Lastly, while the dose reduction from 0.9 mSv to 0.6 mSv achieved by appropriate arm positioning represents an important step in dose optimisation, it may not be the ultimate limit. Data from clinical low-dose chest CTs and post-mortem CTs using ultralow CT tube voltage and tube current settings suggest that the effective dose for MCE CTs might potentially be reduced to 0.2 mSv [10, 35].

## Conclusions

This study examined the effect of arm positioning during CT of the MCE for FAE on radiation dose, scan length, and image noise and found a 33% lower effective radiation dose in arms-up scans compared to arms-down, with no significant difference in scan length or image noise. These findings highlight the importance of arm positioning for radiation protection, particularly in age estimation, where scans are conducted for non-medical reasons on young individuals, and support the practice of performing MCE CT scans for FAE with arms-up.
